# 

**Published:** 1874-07

**Authors:** 


					The Annual Meetings of the Associations.?During the latter
part of the present and the begiitning of the next month, the
annual meetings of the different Dental Associations will take
place; and from present appearances, and the great interest
manifested to secure a large attendance, there is good reason for
believing that these meetings will prove very instructive and
beneficial to all who are fortunate enough to be present and
participate in their proceedings. Below we give the time and
place of meeting of the different organizations:
Southern Dental Association.-?The sixth annual meeting of
the Southern Dental Association will be held Tuesday, the 28th
of July, 1874, in the city of St. Louis, Mo.
Robt. Arthur, M. D., D. D. S., President.
Jas. F. Thompson, M. D., D, D. S., Recording Secretary.
EXTRACT FROM THE CONSTITUTION.
Art. Ill?Membership?Membership in this Association shall
consist of such only as shall have received a degree in Dentistry,
or Medicine, and who is engaged in the practice of Dentistry,
and receives a three-fourths vote of the members present to elect.
Art. XIV?Delegates.?This Association will receive one del-
egate from each Dental Association or Society, and also one
delegate from any corporate Dental Association.
Editorial, Etc. 139
The Executive Committee have made full arrangements to
insure a successful and pleasant gathering.
The printed circular of invitation informs the profession that
the railroads will furnish tickets at a discount of from 10 to 25
per cent., according to number taken, but advises those who can
to get excursion tickets to St. Louis. Twenty per cent, discount
from regular rates may be had at the principal hotels. The
sessions of the Association will be held in Polytechnic Hall,
and Dr. Isaiah Forbes will deliver the address of welcome.
The Niveteenlh Annual Meeting of the American Dental ^4s60-
ciation will be held at Detroit, Michigan, on the 4th of August
next. The following are the principal officers of this Association :
President, T. L. Buckingham, M. D., D. D. S. Recording Secre-
tary, M. S. Dean, D. D. S. Executive Committee, Drs. G. L.
Field, G. R. Thomas and G. H. Cashing.
The Annual Meeting of the American Dental Convention will
as usual, be held at Saratoga Springs, N. Y., on the 11th
of August next. The following are the principal officers of this
organization : President, John Allen, D. D. S. Recording Sec-
retary, C. S. Hurl but, D. D. S. Executive Committee, Drs. Am-
bler, Weed, Gage, Kake and Meritt.
American Academy of Dental Science.?The Seventh Annual
Meeting of the American Academy of Dental Science will be
held in Boston, on Mondav, September 28th, 1874, at 10 o'clock
A. M.
The annual address will be delivered by Dr. W. W. Allport,
of Chicago.
E. N. HARRIS, Corresponding Secretary.
The Annual Meeting of the American Dental Society of Europe
will be held at Geneva, Switzerland, on the 2nd of the present
month?July.
TO READERS AND CORRESPONDENTS.
Communications intended for publication, and exchange journals and papers,
should be addressed to the Editor of the American Journal of Dental,'
Science, 259 N. Eutaw St., Baltimore, Md. All letters relating to the business,
of the journal, or containing cash remittances, to be directedtO Messrs. Snowden^
& Cowman. Articles for publication should be received by the editor at least5
three weeks previous to the first day of the month on which the number in which5
it is designed for them to appear, is issued. j
f^jT?The American Jonrnal of Dental Science will contain fifty pages of
reading matter in each number, making a volume of not less than
Six Hundred pages
EXCLUSIVE OF ADVERTISEMENTS.
THE
AMERICAN JOURNAL
OF
DENTAL SCIENCE
F. J. S. GORGAS, M.D.,D.D.S., Editor.
The Journal is issued on the first of each month and contains not less
than fifty pages of reading matter in each number, exclusive of adver-
tisements, making a volume of six hundred pages per annum.
In each number will be found Original Communications, Corres-
pondence, Selected Articles, Monthly Summary, Bibliographical No-
tices and Editorial Matter, and every effort will be exerted to render
the Journal worthy of the patronage of the Dental profession.
A generous co-operation is respectfully solicited from the members
of the profession ; and as the Journal has now a printing office of its
own which materially lessens the cost of its publication, it will be
issued at the reduced subscription price of
92.SO For Annum in Advanoe.
Communications are respectfully solicited on all subjects pertain-
ing to the practice of dentistry, as well as reports of cases occurring
in dental practice.
RATES OF ADVERTISING.
1 Page.
t "
i "
i ?
1 MO.
$15 00
10 00
7 00
5 00
2 MO.
$22 50
15 00-
11 00
8 00
3 MO.
$30 00
20 00
15.00
11 00
6 MO.
$50 00
30 00
20 00
15 00
12 MO.
$100 00
50 00
30 00
20 00
All original communications designed for publication, works for
review, new instruments for notice, exchanges, &c., should be directed
to the editor, 259 N. Eutaw St., Baltimore, Md.
All advertisements and subscriptions should be addressed to
SNOWDEN & COWMAN,
Publishers of the Am. Journal of Dental Science.
86 W. Fayette St. Bet. Chakles & Libekty &ts.,
BALTIMORE, Md.

				

